# Ion-Pairs in Aluminosilicate-Alkali Synthesis Liquids Determine the Aluminum Content and Topology of Crystallizing Zeolites

**DOI:** 10.1021/acs.chemmater.2c00773

**Published:** 2022-06-10

**Authors:** Karel Asselman, Nick Pellens, Barbara Thijs, Nikolaus Doppelhammer, Mohamed Haouas, Francis Taulelle, Johan A. Martens, Eric Breynaert, Christine E.A. Kirschhock

**Affiliations:** † Center for Surface Chemistry and Catalysis—Characterisation and Application Team (COK-KAT), KU Leuven, Leuven 3001, Belgium; ‡ Institute for Microelectronics and Microsystems, JKU Linz, Linz 4040, Austria; § Institut Lavoisier de Versailles, Université de Versailles Saint-Quentin-en-Yvelines, Versailles Cedex 78035, France; ∥ NMR/X-ray Platform for Convergence Research (NMRCoRe), KU Leuven, Leuven 3001, Belgium

## Abstract

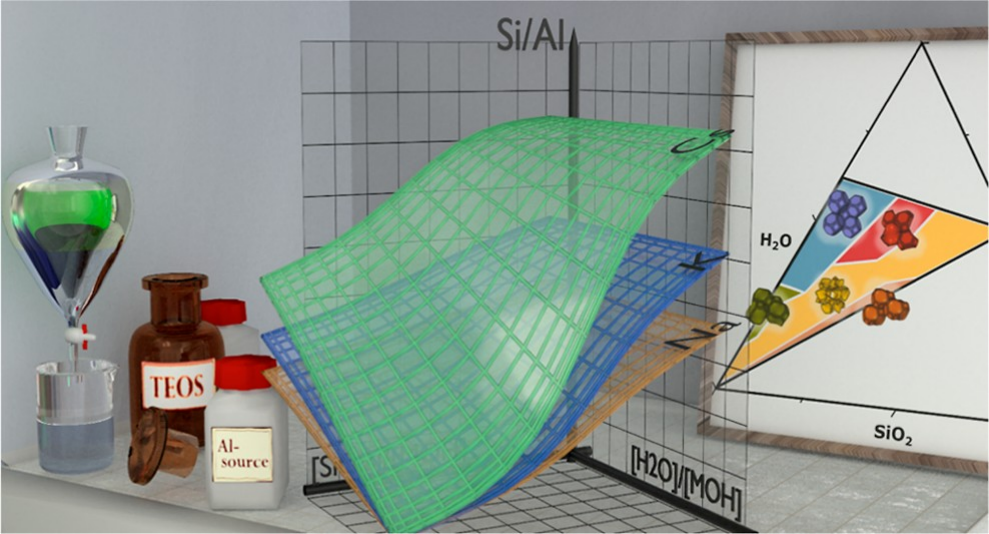

Using hydrated silicate ionic liquids, phase selection and framework silicon-to-aluminum ratio during inorganic zeolite synthesis were studied as a function of batch composition. Consisting of homogeneous single phasic liquids, this synthesis concept allows careful control of crystallization parameters and evaluation of yield and sample homogeneity. Ternary phase diagrams were constructed for syntheses at 90 °C for 1 week. The results reveal a cation-dependent continuous relation between batch stoichiometry and framework aluminum content, valid across the phase boundaries of all different zeolites formed in the system. The framework aluminum content directly correlates to the type of alkali cation and gradually changes with batch alkalinity and dilution. This suggests that the observed zeolites form through a solution-mediated mechanism involving the concerted assembly of soluble cation-oligomer ion pairs. Phase selection is a consequence of the stability for a particular framework at the given aluminum content and alkali type.

## 1 Introduction

For simple minerals in contact with an aqueous solution, clear links between mineral composition and chemical speciation in solution are obvious. Ionic crystals, for example, may directly precipitate from their solvated ions and their formation is easy to predict. In many cases, however, the link between forming solid and dissolved species is far from trivial. The existence of diverse polynuclear species, complex crystallization pathways, interfacial processes, and metastable intermediates often obstructs detailed investigation of crystallization and dissolution.^1,2^ Zeolite formation is a typical example.

Literature suggests zeolite formation is driven by small mobile solution species and their rich polymorphism is governed by an interplay between all framework and pore-filling species during synthesis.^1^ Alkali cations, for example, are empirically known to show preference for specific structural units in the final zeolite.^3^ Furthermore, specific arrangements of alkali cations and their hydration water were identified as structure-directing agents during zeolite formation.^4^ This complexity explains why even small variations of composition and conditions may turn synthesis to vastly different products.^4−8^ Besides the framework topology, zeolite properties are determined by their composition,^9^ characterized for aluminosilicate zeolites by the Si/Al ratio and extra-framework species. Lechert and co-workers^10−13^ were first to empirically establish quantitative correlations between zeolite aluminum content and alkalinity of the liquid phase. The zeolite Si/Al ratio of faujasite (FAU) and some other zeolite topologies such as LTL, RHO, and OFF became predictable, owing to solution speciation models, based on simplified equilibrium distributions of species between solid and liquid fractions of the synthesis gel. Such models, however, rarely address the selection of the framework topology. An exception is the FAU–LTA system where zeolite composition and phase selection could be predicted by a thermodynamic solution model.^14^

In conventional sol–gel synthesis, zeolite formation predominantly takes place in chemically and structurally heterogeneous media, involving complex equilibria and kinetics. Often solid and liquid fractions have not reached steady-state prior to onset of crystallization, as evident from a strong dependency of gel aging time on synthesis outcome.^1,8,15,16^ In such dynamic, multiphasic systems, thermodynamic equilibrium models fall short to predict the speciation in solution, gel, and solid phases, information which is needed to rationalize zeolite properties from the synthesis composition. However, a comprehensive recognition and detailed documentation of relationships between the liquid-state chemistry and stoichiometry with the final crystal appears as an attractive option to improve understanding of zeolite synthesis. Specifically, the impact of synthesis stoichiometry on the resulting zeolite composition, as well as its topology, can be expected to further facilitate the synthesis of zeolites with targeted qualities.

Monophasic hydrated silicate ionic liquids (HSILs), consisting of small, charge stabilized aluminosilicate oligomers, can yield zeolites at highly defined conditions.^17−19^ Owing to severe water limitation, all ions are hypo-hydrated and the liquid is dominated by strong ionic interactions. These ionic liquids closely resemble stable, room-temperature molten silicates. By adding water and/or alkali to the native HSIL, compositions can be varied from severely water-deprived to more dilute systems in a wide range of alkalinity. When doped with aluminate, HSILs can readily yield zeolites in the absence of silicate sol or gel interphases.^17^ In this work, it is shown that the synthesis liquid stoichiometry determines the zeolite Al-content across framework boundaries for various alkali cations and a wide range of compositions. Based on these results, a relation between synthesis stoichiometry, Si/Al ratio of a zeolite, and resulting framework topology is proposed.

## 2 Experimental Section

### 2.1 Sample Preparation

Zeolite samples are synthesized using the reported method via HSILs.^17,18^ HSILs are prepared by mixing tetraethyl orthosilicate (TEOS), alkali hydroxide, and deionized H_2_O into an emulsion with molar ratios listed in Table S1 under continuous agitation. After complete hydrolysis of TEOS into silicic acid and ethanol, spontaneous liquid–liquid phase separation results in an upper water–ethanol phase, and a dense, inorganic HSIL phase, which is collected via a separation funnel. Final HSIL compositions, listed in Table S2, were determined via gravimetric analysis.^17^

**Figure 1:**
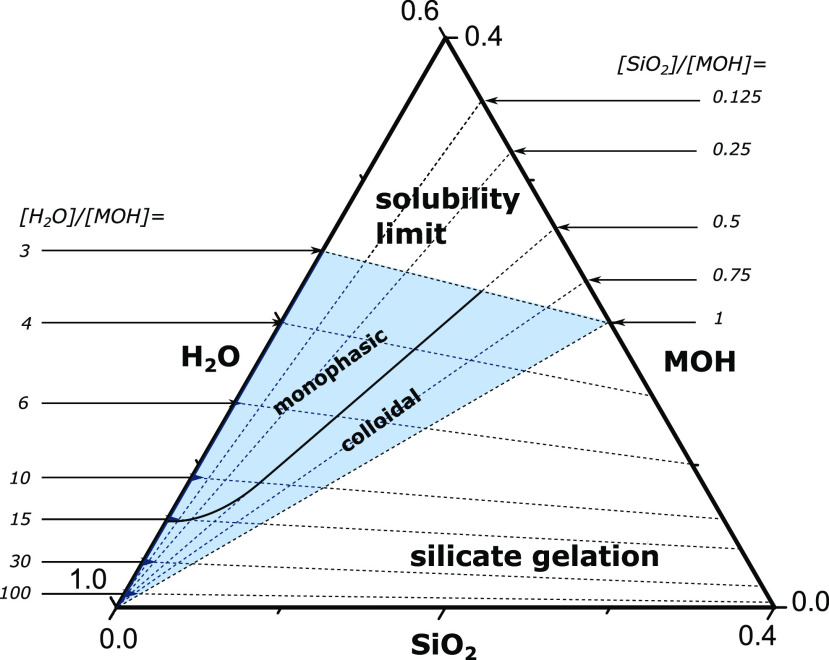
Representation of the ternary phase diagram. The blue area indicates the explored chemical space. Batch compositions were chosen at intersecting lines of discrete alkalinity and cation hydration values. Extra compositional points were added for each cation system independently to better discern the phase boundaries (Figure S4 and Table S3). The indicated boundary separates regions which form homogeneous true liquids or colloidal suspensions upon aging after the addition of aluminate. The precise position of the boundary varies only slightly when the alkali cation is changed.

Starting from a native HSIL, the desired synthesis composition is obtained by adding water and alkali hydroxide (MOH). The SiO_2_–MOH–H_2_O ternary diagram shown in Figure 1 illustrates the accessible chemical space, limited by the solubility of alkali silicate ([H_2_O]/[MOH] = 3) and the liquid/gel phase boundary ([SiO_2_]/[MOH] = 1). A ratio of [SiO_2_]/[MOH] > 1 (low alkalinity) destabilizes the liquid due to insufficient silicate deprotonation, resulting in silicate polymerization.^17^ By subsequent doping with aluminate, synthesis liquids in the composition window of 0.5SiO_2_/0.013Al_2_O_3_/*x*MOH/*y*H_2_O were prepared in the presence of Na, K, and Cs alkali cations with a constant and high SiO_2_/Al_2_O_3_ ratio of 40. All molar synthesis stoichiometries are listed in Table S3 and indicated in the ternary diagram representation in Figure S4. The liquids were stirred for 24 h at room temperature and then hydrothermally treated in Nalgene Oak Ridge PPCO centrifuge tubes (Fischer Scientific) at 90 °C for 7 days in a tumbling oven. After synthesis, samples were recovered, and the crystals were separated from the mother liquor and washed by repeated dispersion-centrifugation. The solids were subsequently dried at 60 °C and stored under ambient conditions prior to further characterization.

### 2.2 Characterization of Synthesis Products

High-resolution scanning electron microscopy (SEM) images were recorded with a Nova NanoSEM450 (FEI, Hillsboro, OR). For chemical analysis, Si and Al contents were determined on an axial simultaneous ICP–OES instrument (Varian 720-ES) with cooled cone interface and oxygen-free optics. Samples for ICP were prepared by digesting 50 mg of zeolite powder with 250 mg of LiBO_2_ in a muffle furnace at 1000 °C prior to diluting with 0.42 N HNO_3_ solution. Laboratory high-throughput powder X-ray diffraction (XRD) patterns were recorded at room temperature on a STOE STADI P Combi diffractometer (Cu Kα radiation), with focusing Ge(111) monochromator and a 140° curved image plate position sensitive detector. XRD patterns of all samples, chemical analysis, and selected SEM images are available in the Supporting Information (Figures S1–S3 and S5 and Table S3). Samples are labeled according to the cation hydration [H_2_O]/[MOH] and batch alkalinity [SiO_2_]/[MOH] of the synthesis mixture. For example, the label MOH A–B indicates a zeolite formed in a synthesis mixture with respective cation hydration and alkalinity of A and B.

## 3 Results

### 3.1 Room-Temperature Sample Stability

At room temperature, upon the addition of aluminate, all mixtures with [SiO_2_]/[MOH] < 0.5 and [H_2_O]/[MOH] < 15 form monophasic, optically clear liquids.^20^ This region, characterized by high alkalinity and low water content, yields hypo-hydrated ionic liquids.^17,20^ In these liquids, high charge density stabilizes aluminum in small aluminosilicate oligomers through intimate interactions with alkali cations, leading to aluminate solubility much higher than previously predicted by aluminosilicate speciation models,^12,20−23^ similar as observed for the increased solubility of gibbsite in concentrated electrolytes.^24^ With the increase in dilution, ionic stabilization of small aluminosilicate oligomers decreases, facilitating their condensation into larger aluminosilicate species with a lower solubility, therefore, triggering the formation of aggregated aluminosilicate species, manifesting as colloids^20,25^ (Figure 1). A similar trend is observed with the decrease in alkalinity, owing to reduced charge density and ionic stabilization. After the removal of these aggregates by centrifugation (15 min, 35,000*g*), the liquids remain stable for extended periods of time. This confirms the observed colloids arise from the limited solubility of aluminosilicate species (Table S4), and polymerization leading to gelation occurs only after the depletion of hydroxide ions or introducing excess cationic aluminum.

### 3.2 Characterization of Synthesis Products

**Figure 2:**
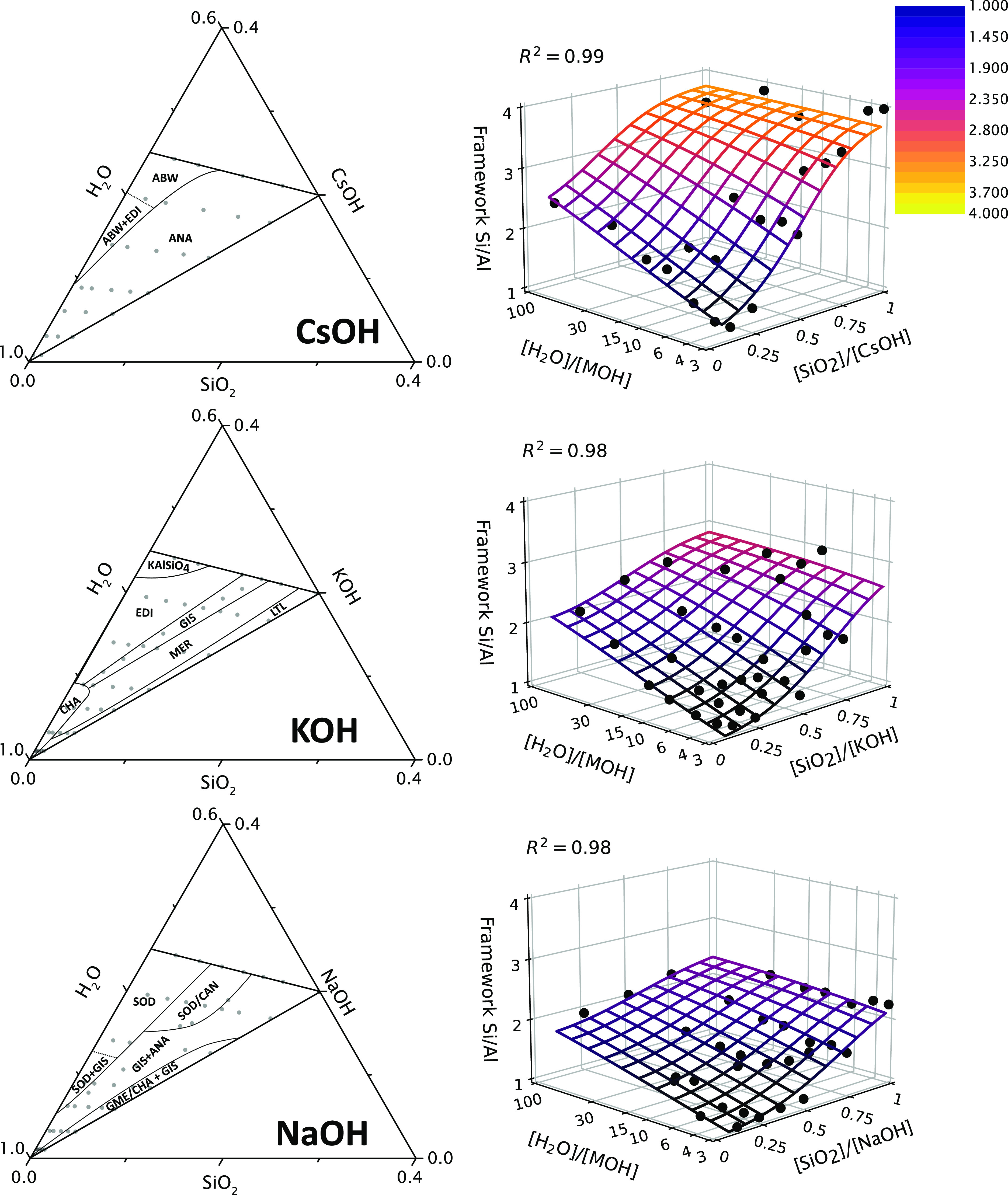
(left) Frameworks formed by batch composition in qualitative ternary diagram representation. A “+”-sign indicates a phase mixture, while a “/”-sign indicates an intergrowth of two frameworks. The compositions of synthesized samples illustrated in the ternary diagrams (gray points). (right) Framework Si/Al ratio represented as a function of batch alkalinity [SiO_2_]/[MOH] and batch cation hydration [H_2_O]/[MOH]. Phase selection as function of alkalinity and cation hydration is illustrated in Figure S4.

Upon hydrothermal treatment, the synthesis liquids yield various zeolite frameworks, as indicated in the ternary phase diagram (Figures 2 and S4). The framework boundaries were derived from a total of 121 samples spread evenly across the ternary diagrams. A total of 11 different framework types were identified: ABW, ANA, CAN, CHA, EDI, GIS, GME, LTL, MER, and SOD, along with kalsilite (KAlSiO_4_), a non-porous potassium aluminosilicate. Some topologies are unique for a certain cation system, while others can crystallize with multiple cations. For instance, GIS forms with Na or K, ANA with Na or Cs, and EDI with K or Cs. Interestingly, this is the first report of GIS-type zeolite crystallizing directly from purely K-based synthesis mixtures. Figure 2 and Table S3 document the framework composition of formed zeolite products. For each cation type, the Si/Al ratio varies smoothly and continuously with batch alkalinity and water content, with no discontinuities at the observed phase boundaries. For constant [H_2_O]/[MOH], the framework Si/Al ratio continuously increases with the decrease in alkalinity (i.e., increasing [SiO_2_]/[MOH]). Likewise, for constant and high alkalinity, increasing water content also increases the Si/Al ratio in zeolites produced. With the decrease in batch alkalinity, the effect of increasing hydration diminishes to finally become negligible for [SiO_2_]/[MOH] = 1. Extending previously derived relations between batch stoichiometry and zeolite Si/Al ratio;^10−13^ here, it is observed that the alkali cation type is a major determinant for the framework aluminum content. For each cation type, zeolites with the highest possible aluminum content, that is, Si/Al = 1, form at the highest alkalinity and lowest water content. The increase of framework Si/Al with the decrease in batch alkalinity and increase in water content, respectively, is much steeper for larger cations. With values ranging from 1 up to 4, the increase of the Si/Al ratio of the zeolite with dilution and the decrease in batch alkalinity is most pronounced for Cs-zeolites. For K- and Na-zeolites, the Si/Al ratio increases to approximately 3 and 2.2, respectively.

While the smooth evolution of the framework Si/Al ratio with batch composition shows a similar trend for all three cations, the correlation between the framework composition and framework topology markedly differs. For all cations, the highest alkalinities and lowest water contents always give rise to a framework Si/Al ratio of 1 but manifest in zeolites with different framework topologies (Na-SOD and K-EDI or kalsilite and Cs-ABW). These frameworks are known for their high stability at this Si/Al ratio,^26^ as well as for their high selectivity for the respective alkali ion.^27−29^ Zeolite phase selection in the K-system closely correlates to the observed framework aluminum content as the obtained frameworks (Figure 2) exist within a relatively narrow range of possible framework aluminum contents and are known to be stable for the given Si/Al framework ratio (Table 1). In the Cs^+^-system, the analcime framework (ANA), known for its compositional flexibility, forms over a wide range of batch compositions with framework Si/Al ratios ranging from 1.8 up to even 4. Sodium, similar to potassium, yields many different topologies, including intergrowths and phase mixtures, again coinciding with typical Si/Al ratios of the formed frameworks (Table 1). Similar to the cesium system, some span a wider region, such as the GIS and ANA biphasic region, found over a wide range of alkalinity and water content.

**Table 1:** Framework Compositions of This Work Compared to Those Reported in Literature^a^

framework	cation type	Si/Al (this work)^b^	Si/Al(literature)	references
ABW	Cs	1.14–1.33	1	(30−32)
	Li, Rb		1	(30) and (33)
GIS	Na	1.26–2.25	1–3.44	(34−39)
	K	1.18–1.44		
ANA	Na	1.47–1.7	1.47–3.10	(40−42)
	Cs	1.76–3.98	2–2.4, 4.11^c^	(42−44)
	K		2	(45)
CHA	K	1.65–1.82	1.4–2.67	(42) and (46−49)
LTL	K	2.47–2.96	2.3–3.5	(50−54)
MER	K	1.64–2.58	1.70–2.36	(18), (42), (55), and (56)
	Rb		3.77	(57)
SOD	Na	0.99–1.04	1	(58−60)
EDI	K	1.03–1.22	1–1.5	(29) and (61−63)
	Cs	1.15	1	(64)
	Li		1	(65)
kalsilite	K	1.08	1	(66)
SOD/CAN^d^	Na	1.08–1.11	1	(67) and (68)

a Reference materials (synthetic or natural) with a single-type alkali cation are listed for comparison.

b Si/Al values of samples with high phase purity (estimated from XRD) were added to the table.

c Isolated occurrence with unusually high framework Si/Al.

d This frequently occurring intergrowth phase is commonly denoted in literature as INT-phase.

Remarkably, independent of zeolite topology, phase purity, or the occurrence of intergrowths, framework Si/Al follows a continuous trend with respect to the synthesis liquid composition. While phase boundaries are relatively sharp and markedly differ between cations, the Si/Al ratio evolves smoothly. To investigate this surprising observation in detail, zeolite crystallization was evaluated as a function of time for a Na-based synthesis composition producing a 50/50 mixture of GIS and ANA after 7 days (Figure S6). The recrystallization kinetic of the GIS phase into the thermodynamic ANA phase is a commonly observed transformation, formation of the latter being promoted by higher synthesis temperature and/or longer crystallization time.^7^ In the series, initially GIS formed, while over time, the fraction of ANA gradually increased. Irrespective of the relative amounts of the two frameworks, the Si/Al ratio remained unchanged at 1.39  0.15, demonstrating that during the here observed framework conversion, the zeolite framework Si/Al ratio did not change and is determined by the initial batch stoichiometry, regardless the framework topology. It, therefore, appears that the framework Si/Al ratio is not thermodynamically determined.

Comparison of synthesis outcome in the absence or presence of colloidal aggregates addresses the role of the liquid and solid phases during zeolite nucleation and growth. None of the ternary diagram systems show discontinuities neither in the framework topology nor aluminum content when crossing from the monophasic liquid regime to the region where colloidal aggregation is observed (Figure 2). This indicates that the presence of colloids does not influence the synthesis outcome as long as the liquid composition does not considerably change, that is, when the fraction of removed solids is small. For further confirmation, the synthesis of selected samples was repeated using only the liquid phase after removal of the colloidal aggregates by centrifugation. Departing from the hypothesis zeolite formation is a solution-driven process, and this should not affect nucleation and growth of the zeolite phase. As expected, removing the solids had negligible impact on the synthesis outcome. Details on these syntheses are added to Supporting Information, Section S2.

## 4 Discussion

The results suggest that the incorporation of aluminate in zeolite frameworks is determined by the state of the synthesis liquid, regardless of which topology is observed. Considering aluminate to be the limiting parameter in the chosen compositional range, it can even be stated that the chemical environment of liquid-state aluminum determines the zeolite framework Si/Al ratio. In extension, this suggests that framework selection is the result of the stability of the topology at this given Si/Al ratio and not vice versa.

Liquid-state ^27^Al NMR measurements have shown that the aluminate speciation in HSIL synthesis liquids exclusively consists of small aluminosilicate oligomers, and zeolites crystallize from these liquids upon the supersaturation of specific aluminosilicate oligomers.^20^ Therefore, it is possible to rationalize our observations by considerations of aluminosilicate solution chemistry.^1,69^ Increasing chemical activity of hydroxide promotes depolymerization of aluminosilicates via deprotonation and hydrolysis, increasing the oligomer charge density and stabilizing oligomers of low nuclearity, including the Al-containing species.^70^ Inversely, decreasing deprotonation, that is, increasing [SiO_2_]/[MOH], leads to polymerization into larger aluminosilicate species. The here described systems with high charge density strive for maximum distribution of aluminate centers at the given degree of condensation.^71^ Hence, the presence of larger aluminosilicate oligomers can increase the Si/Al ratio of the liquid aluminosilicate species by increasing numbers of silicon centers per aluminum. Additionally, in liquids of high charge density, aluminosilicate oligomers are already in close ionic interactions with charge-balancing alkali cations.^20,71,72^ NMR investigations of McCormick et al.^73−76^ showed that increasing alkali cation size leads to a stronger interaction with larger silicate oligomers. As a consequence, larger cations shift the oligomer distribution toward larger species.^74,76,77^ Cation hardness also determines the hydration energy, which relates to the affinity to water coordination.^78−80^ Therefore, changing strongly hydrophilic sodium cations for softer cesium cations with high affinity for silica affects the nature of aluminosilicate oligomers which are in close interaction with the cation via ion-pairing. As the alkali cation affinity to interact with aluminosilicate oligomers directly affects their spatial aluminate distribution and geometry of the aluminosilicate organization in the liquid state, the large impact of cation-type on the observed zeolite Si/Al ratio can be evaluated from their liquid-state stability. For example, cesium, a soft cation with hydrophobic affinities,^81^ can maximize its interaction with silica by pairing preferentially to larger oligomers with a low charge density (higher Si/Al). This effect is directly reflected in the observed zeolite framework Si/Al ratio formed with different cations at otherwise identical batch compositions. The here observed trends over a wide range of batch compositions and zeolite topologies suggest cation-oligomer interaction within the liquid phase already has significant impact, even before nucleation and condensation onto a growing zeolite surface.

Changing water content could also affect the deprotonation state of the oligomers. pH measurements^20^ show, however, that in HSIL systems, the hydroxide activity does not significantly change with the increase in water content, resulting in unchanged average deprotonation state at given [SiO_2_]/[MOH]. Still, a strong effect of water content on the product Si/Al ratio is observed here (Figure 2). This is rationalized by a decrease of the global charge density in the system with the increase in cation hydration, that is, a charge dilution effect. With the decrease in water content, the number of charges per volume increases, favoring close cation interaction with aluminosilicate species via the formation of ion pairs.^20,82^ With the increase in oligomer size and connectivity, their charge densities decrease due to siloxane bridge formation. Therefore, increasing ionic interactions aids the reduction of oligomer size.^20^ This effect is most pronounced at high ion concentrations determined by the number density of the cation, that is, low values of [H_2_O]/[MOH] and [SiO_2_]/[MOH], explaining both the flattening of the planes (Figure 2) at low alkalinity and at high hydration.

Still, the question remains which factors govern the selection of a specific framework topology. Computational^26^ and experimental^83,84^ studies show that the energetic stability of aluminosilicate frameworks for each individual topology depends on the aluminate content, implying every framework has a favorable framework Si/Al ratio range. Framework energy plays a critical role in determining possible pathways of solidification.^85^ While literature does not report typical aluminum contents for all topologies synthesized here, simulations^26^ show that SOD, EDI, and ABW are most stable with the highest possible aluminum content, that is, Si/Al ratio = 1. Likewise, some frameworks were predicted to be energetically stable for a wider range of aluminate incorporation. ANA is such an example, readily forming in the Na^+^ and Cs^+^ systems with Si/Al ratio ranging between 1.8 and 4. In the K^+^ system, merlinoite (MER) is obtained at Si/Al ratios between approximately 1.7 and 2.6, again being the typical Al contents reported for this framework (Table 1).

For decades, it has been observed that zeolite polymorphism and zeolite transformations are determined by cation type and the Ostwald step rule.^1,7,46,86^ Furthermore, for a given cation type, zeolite transformations usually follow specific schemes with respect to time and temperature, for example, FAU to GIS to ANA and LTA to SOD to CAN. These observations, as well as the preference for specific topology with targeted Si/Al ratio for a given cation, were interpreted by the stabilization of (partially) hydrated extra-framework cations acting as template species. These templating cations were often thought to attach to the growing crystal surface independently and induce reorganization of either an amorphous layer of framework species or small oligomers from solution into the most stable configuration.^1,12^ Provided that ionic interactions between cations and aluminosilicate oligomers are already dominantly present before the onset of nucleation,^20^ zeolite formation and transformation can instead be seen as a supramolecular organization, simultaneously incorporating framework and extra-framework species from solution in a concerted assembly. These oligomer–cation interactions are necessary to stabilize viable building units in the solution and also to facilitate their polycondensation on the growing crystal (templating effect).^71,72^ This view of zeolite growth as a self-assembly of cation-stabilized “packing units” (small cages or rings) has been proposed earlier in theoretical studies to predict (un)feasibility of formation of hypothetical zeolite structures.^87,88^ Larger and more complex cages or composite building units are unlikely to be stable in solution and have indeed not yet been observed experimentally, but can be readily assembled at the crystal surface by smaller, mobile liquid species that are in ionic interactions with participating cations.^20,87,89^ Recently, a zeolite crystallization model based on in situ conductivity measurements has shown that the cation type plays a crucial role in the kinetics of this assembly process.^86^

Consequently, coordination environments of cations and framework in the final zeolite should reflect the local chemistry and arrangements within the solution. In this work, this is observed by the fact that the hydrophobic nature of Cs in solution is respected in the ANA and ABW frameworks, where Cs is immobile and fully coordinated to framework oxygen, and not by pore water. In analogy, concentrated NaOH mixtures wherein hydroxysodalite crystallizes (Figure 2), a clear correspondence exists between the coordination assemblies of sodium clusters in the synthesis liquid and within the sodalite cages after crystallization.^4^ Therefore, it can be suggested that zeolite polymorphism is, similar to zeolite composition, a direct result of the chemical state of the synthesis liquid prior to nucleation.

## 5 Conclusions

In summary, this work demonstrates the aluminum content of a zeolite framework synthesized from HSIL media to be a direct function of the synthesis liquid composition. The relation is valid across framework boundaries in the ternary diagrams correlating synthesis composition to topologies. This link between synthesis liquid stoichiometry, zeolite composition, and polymorphism is proposed to arise from a crystallization pathway based on supramolecular assembly of small aluminosilicate oligomers already in intimate interaction with alkali cations in solution. This hypothesis implies zeolite polymorphism and composition to be fully determined by the liquid speciation preceding crystal nucleation and growth. General applicability of this hypothesis needs to be confirmed, but the relation and the experimental observations hold potential for the rational design of functional zeolite materials with targeted properties.

## Supporting Information Available

XRD patterns of all samples, chemical analysis data, molar compositions of synthesis mixtures, selected SEM images, and experimental details of the colloid-free syntheses (PDF)
