# Supplementary material for: Ion-Pairs in Aluminosilicate-Alkali Synthesis Liquids Determine the Aluminum Content and Topology of Crystallizing Zeolites
Source: Chem Mater. 2022 Jun 10;34(16):7150–8. doi: 10.1021/acs.chemmater.2c00773 (PMC9404546; doi:10.1021/acs.chemmater.2c00773)
Supplement: Supplementary file 1 — cm2c00773_si_001.pdf [file cm2c00773_si_001.pdf]

SUPPLEMENTARY INFORMATION TO:

Ion-pairs in aluminosilicate-alkali synthesis liquids determine aluminium content and topology of crystallizing zeolites

Karel Asselman[1†], Nick Pellens[1†], Barbara Thijs[1], Nikolaus Doppelhammer[1,2], Mohamed Haouas[3], Francis Taulelle[1,4], Johan A. Martens[1,4], Eric Breynaert[1,4,*], Christine E.A. Kirschhock[1]

[1]Center for Surface Chemistry and Catalysis – Characterisation and Application Team (COK-KAT), KU Leuven, 3000 Leuven, Belgium
[2]Institute for Microelectronics and Microsystems, JKU Linz, 4040 Linz, Austria
[3]Institut Lavoisier de Versailles, Université de Versailles Saint-Quentin-en-Yvelines, 78035 Versailles Cedex, France
[4]NMR-Xray platform for Convergence Research (NMRCoRe), KU Leuven, 3000 Leuven, Belgium

[†]K. A. and N.P. contributed equally to this work
*Corresponding author: eric.breynaert@kuleuven.be

1. Supplementary figures and tables

Table S1: Nominal molar composition of HSIL formulations (prior to phase separation).

	TEOS	H_2O	MOH
Na	1	26.4	1
K	1	20.8	1
Cs	1	9.6	1

Table S2: Nominal molar composition of HSIL-phases (after coacervation and removal of water-ethanol phase).

	H_4SiO_4	H_2O	MOH
Na	1	2.1	1
K	1	3.6	1
Cs	1	2.36	1

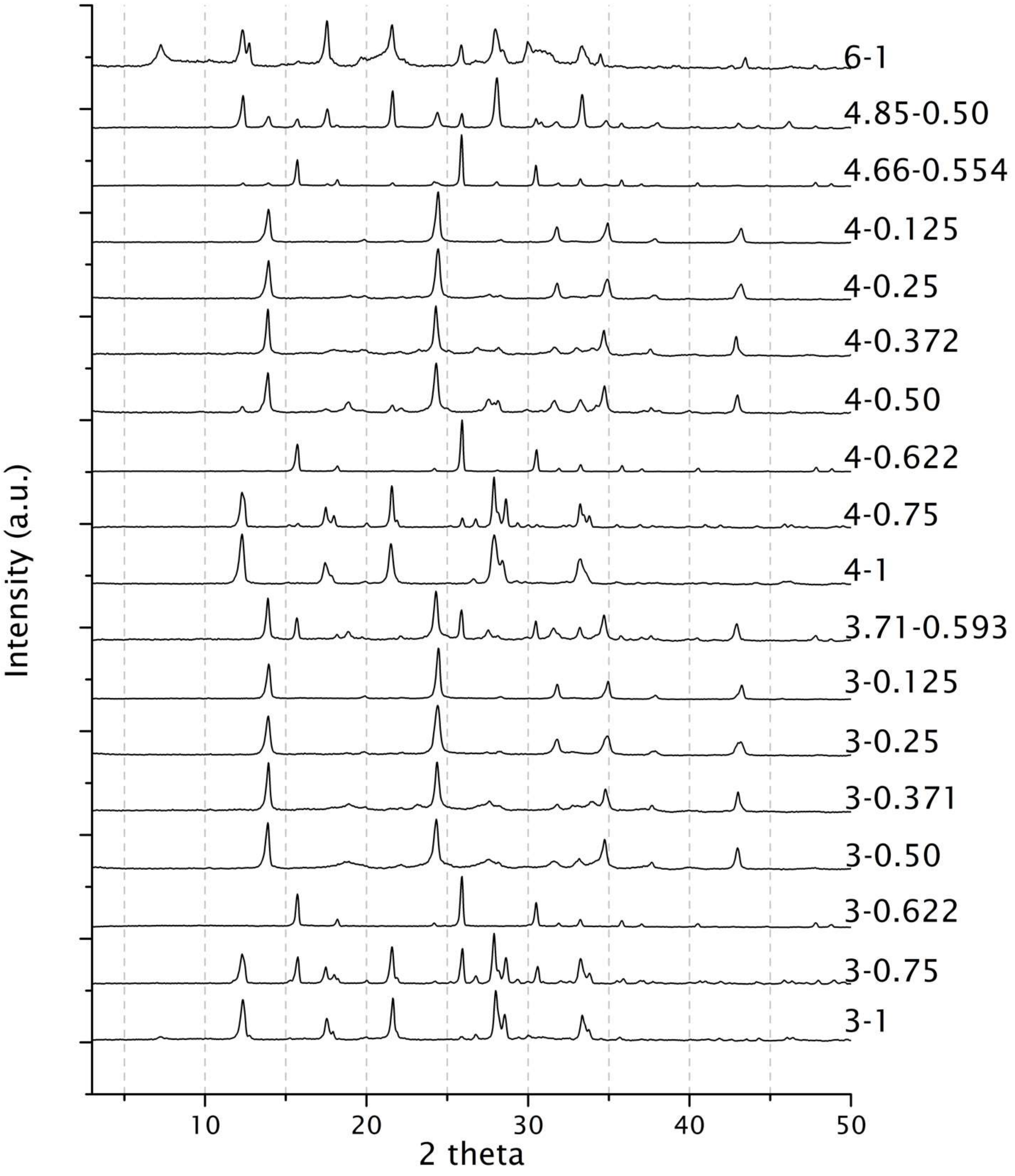

Figure S2: PXRD patterns of sodium-based syntheses.

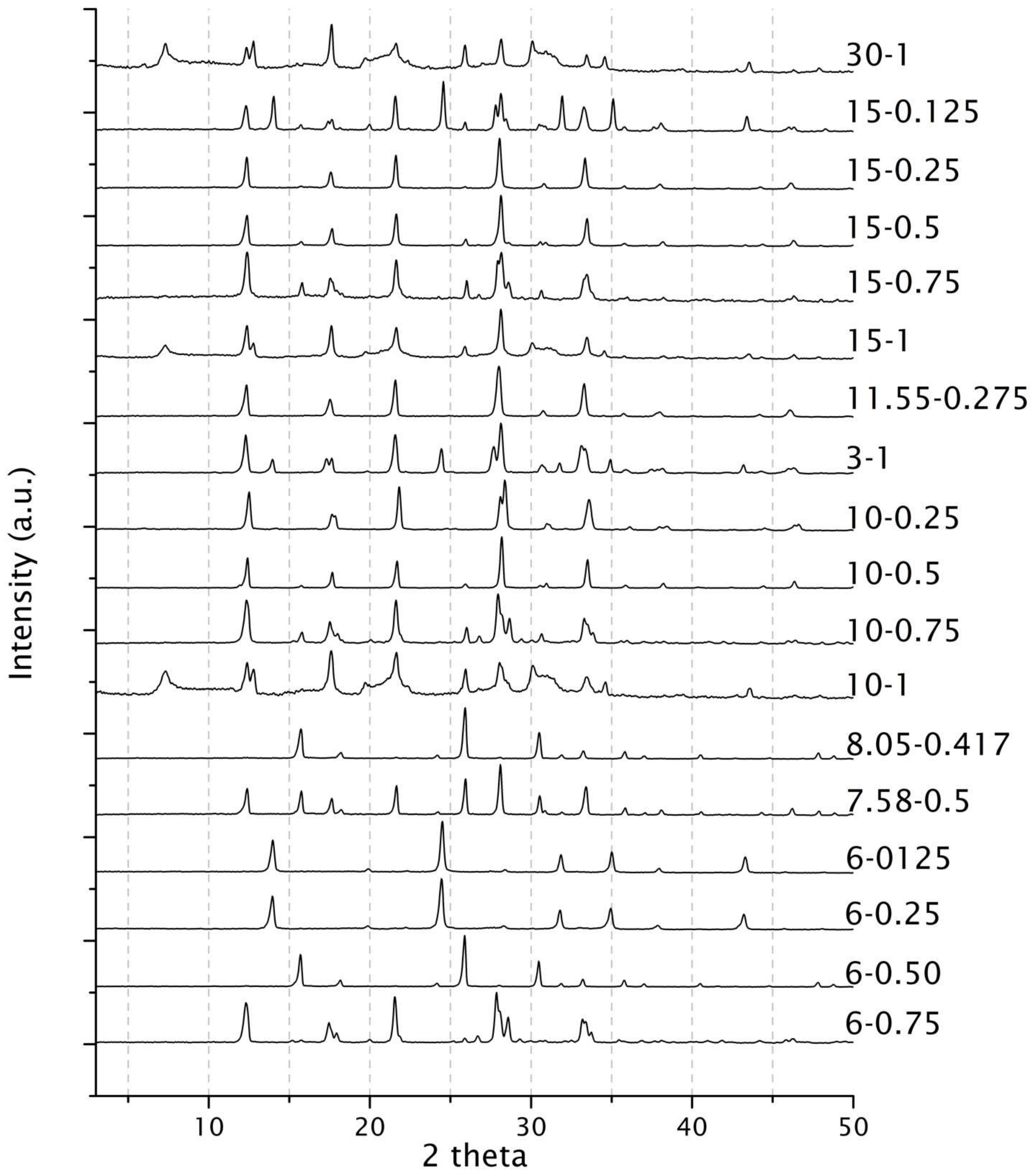

Figure S2 (ctd.): PXRD patterns of sodium-based syntheses.

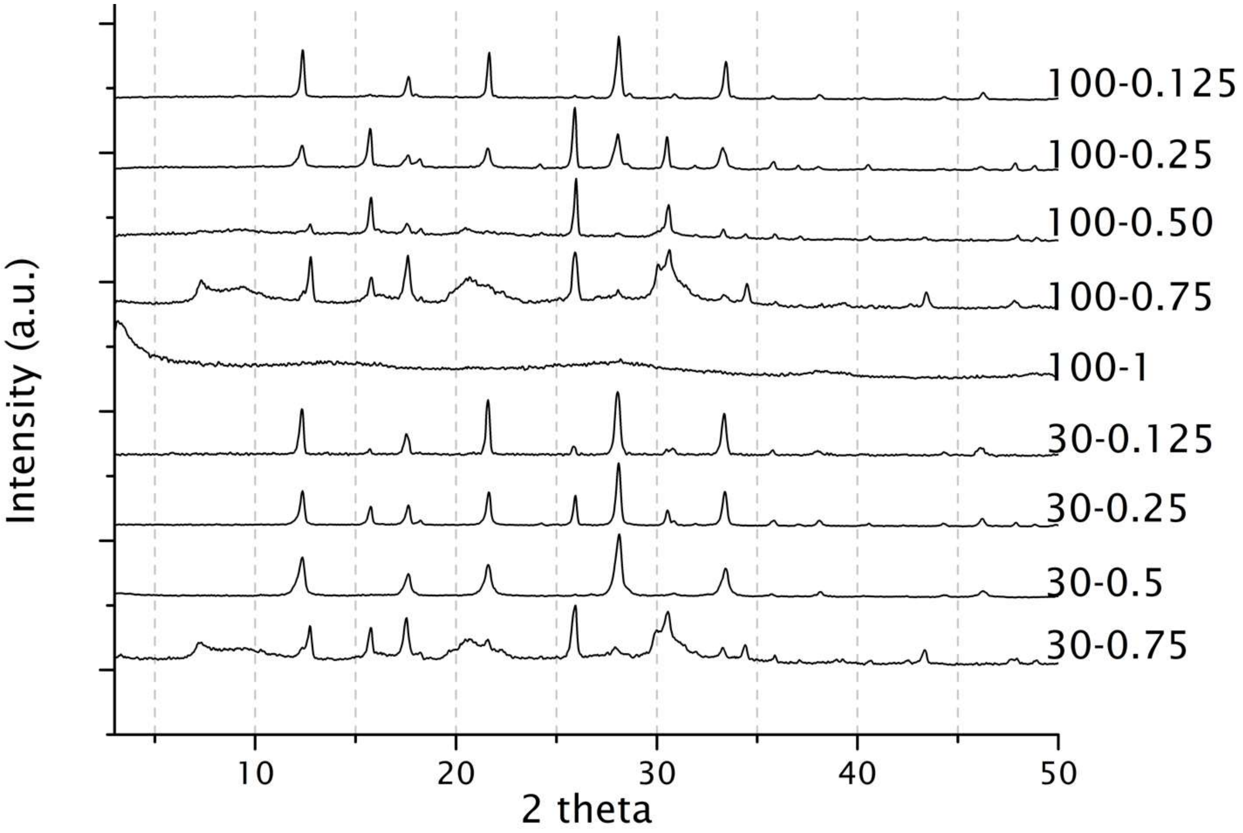

Figure S2 (ctd.): PXRD patterns of sodium-based syntheses.

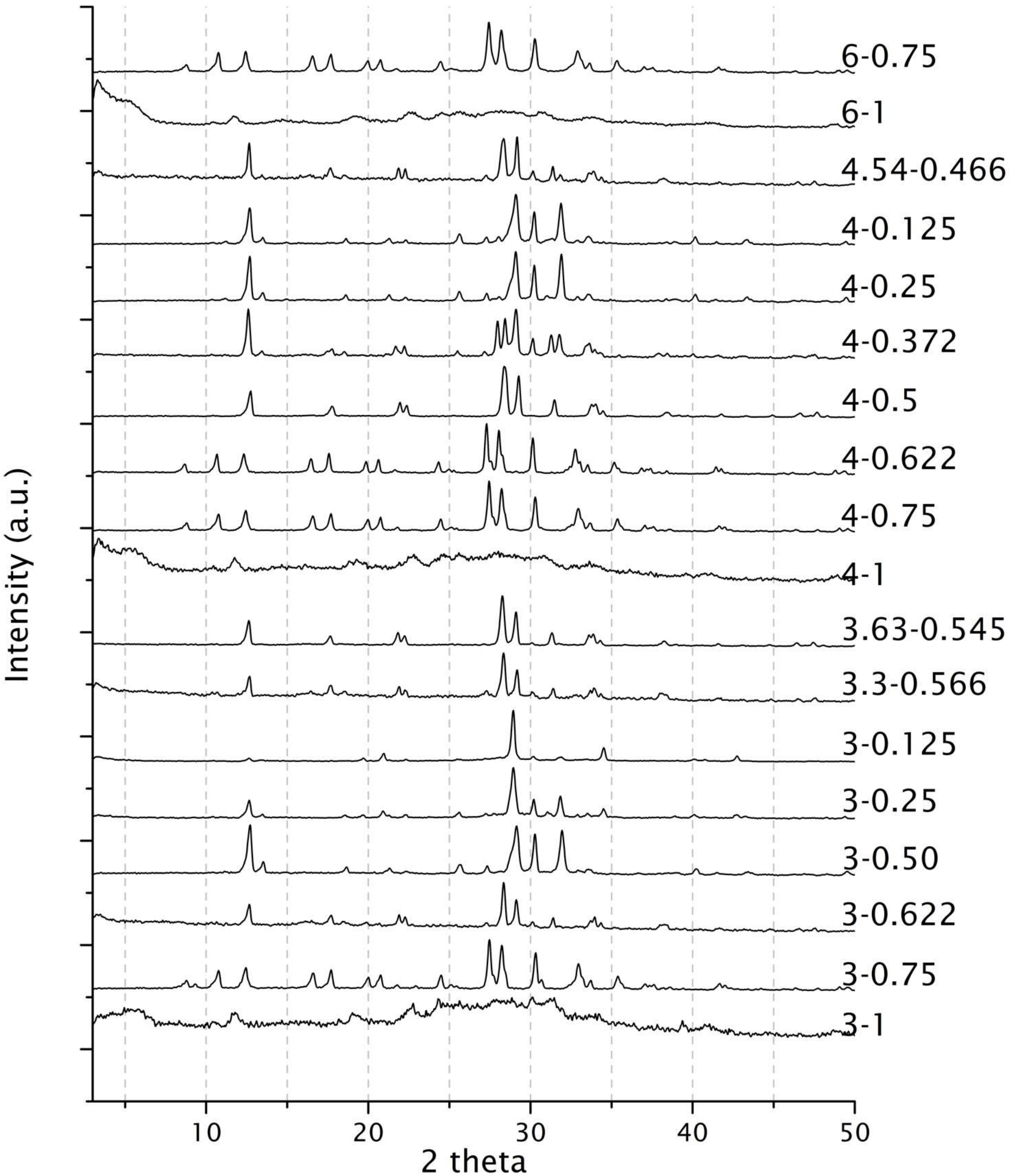

Figure S1: PXRD patterns of potassium-based syntheses.

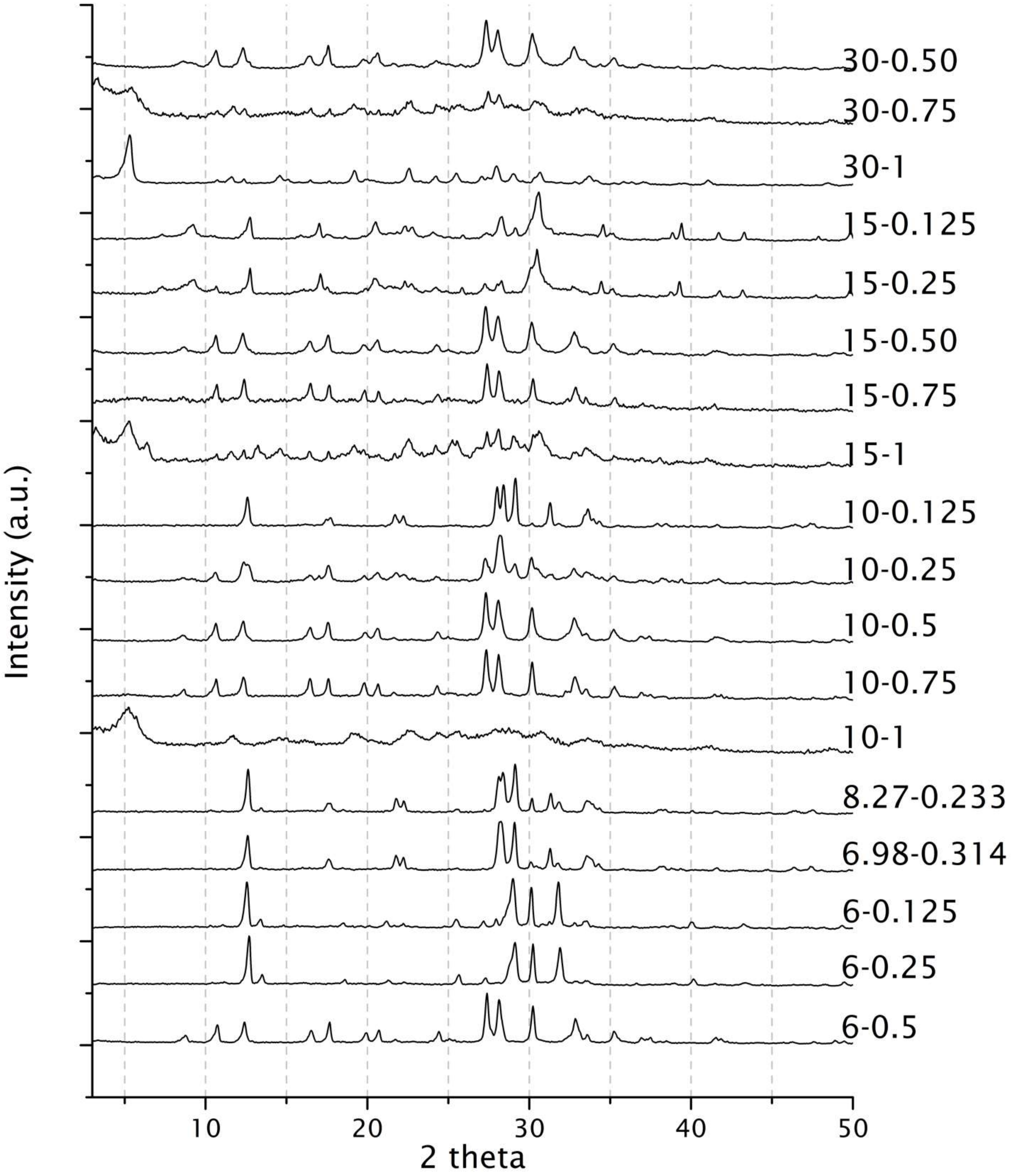

Figure S1 (ctd.): PXRD patterns of potassium-based syntheses.

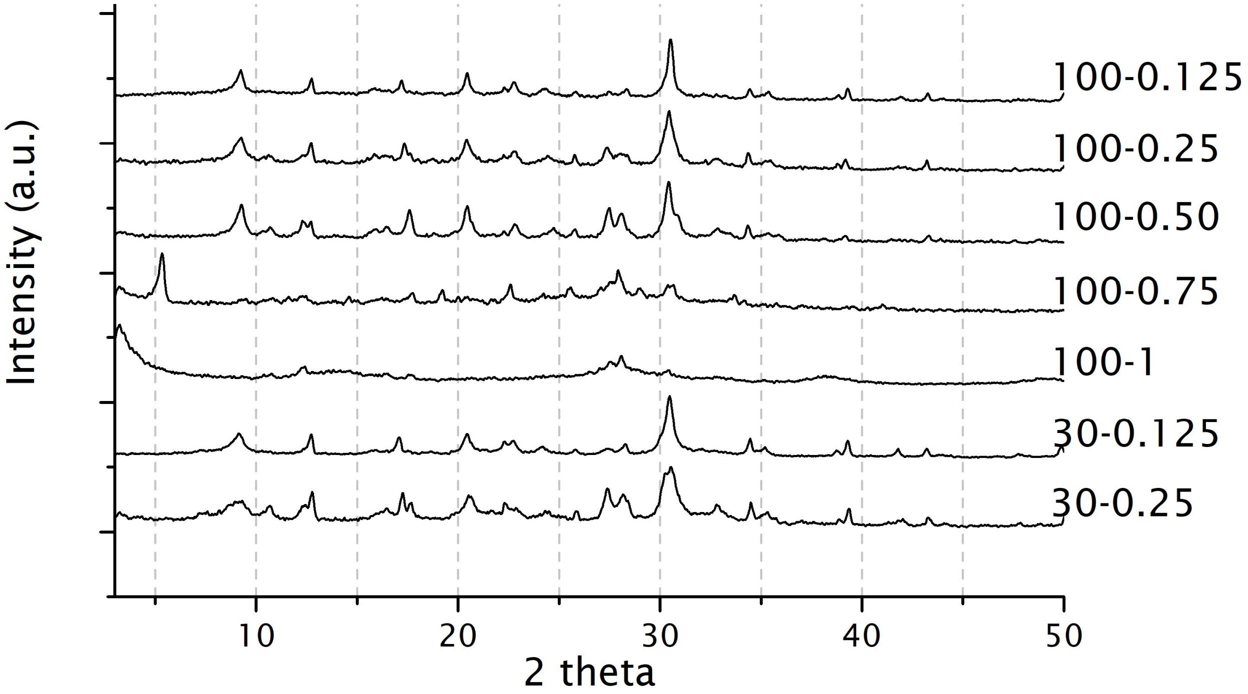

Figure S1 (ctd.): PXRD patterns of potassium-based syntheses.

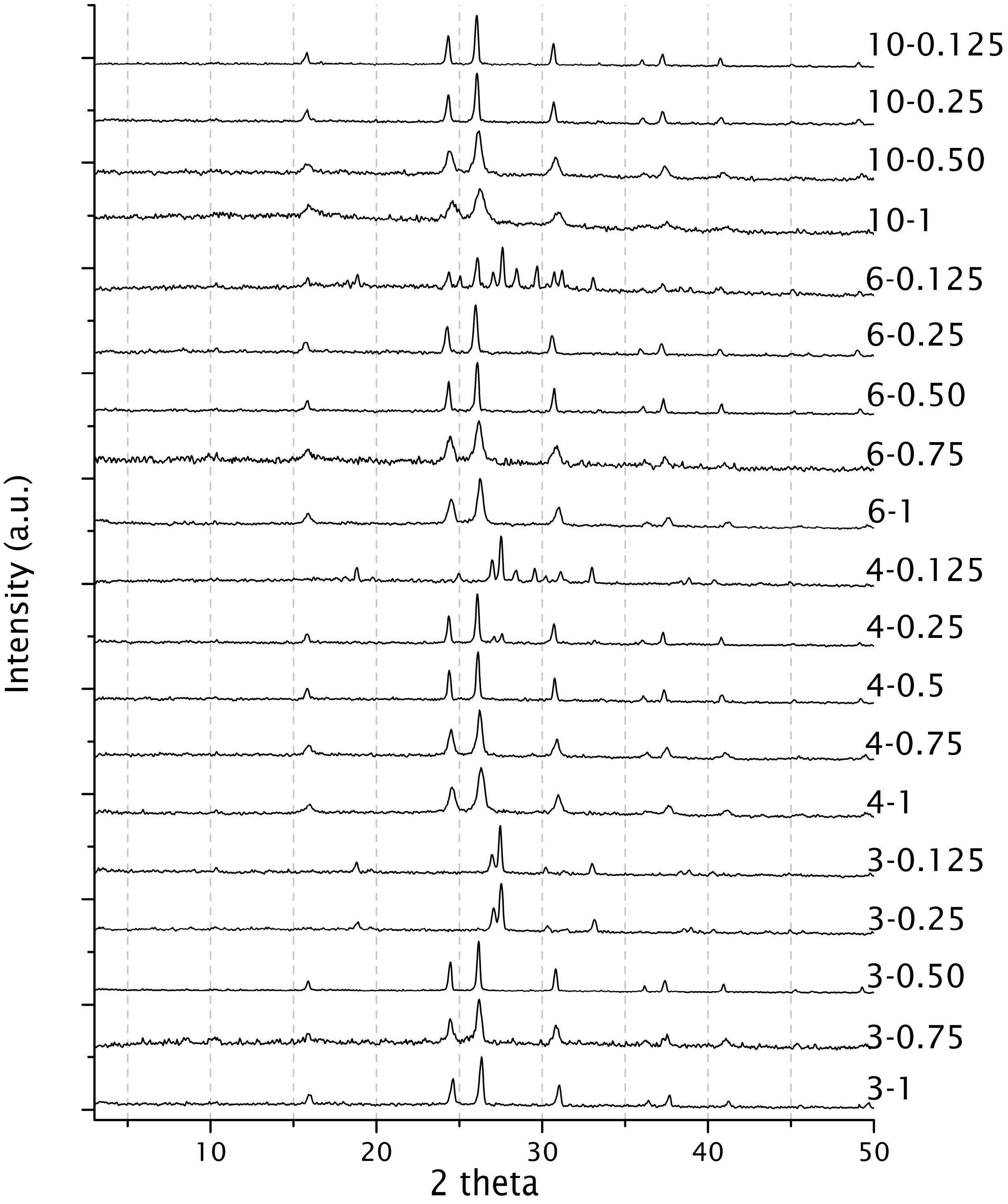

Figure S3: PXRD patterns of cesium-based syntheses.

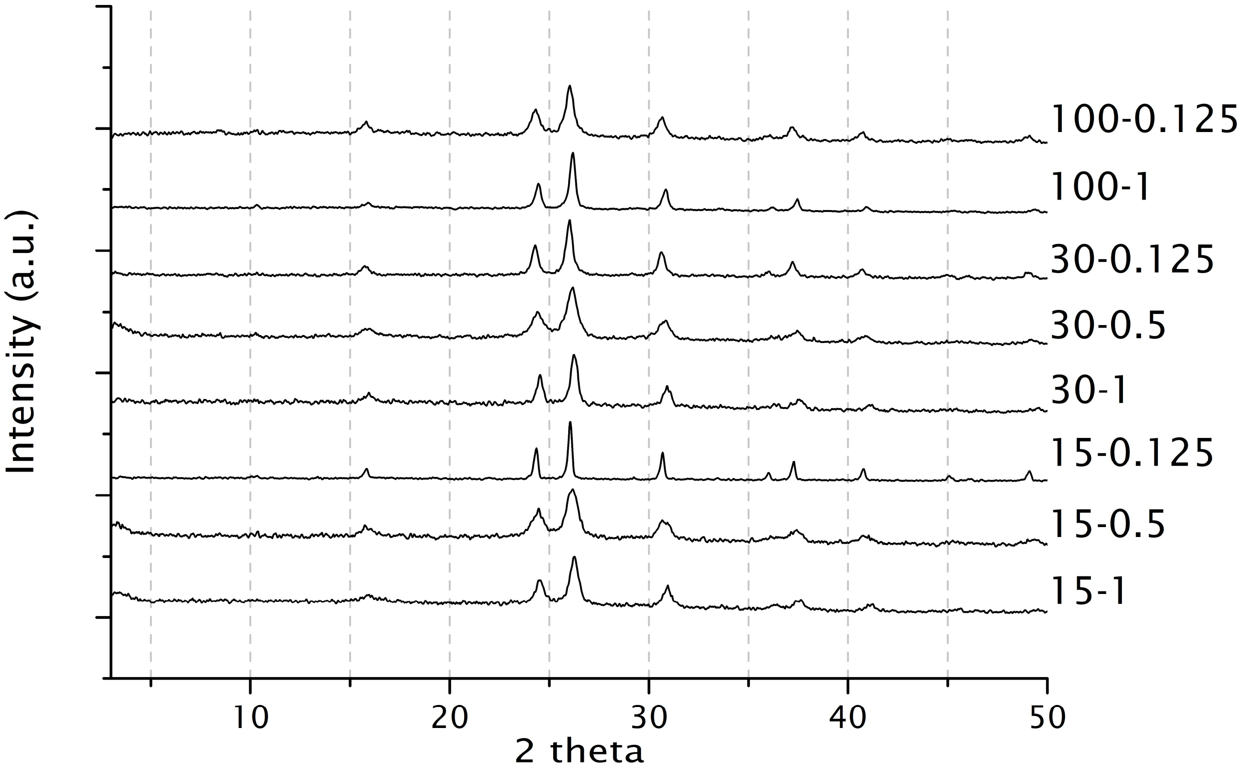

Figure S3 (ctd.): PXRD patterns of cesium-based syntheses.

Table S3: molar compositions of synthesis mixtures.

a. Sodium-based mixtures

Label		Molar composition			
H_2O/NaOH	[SiO_2+Al_2O_3]/NaOH	SiO_2	Al_2O_3	NaOH	H_2O
3.0	0.125	0.5	0.013	4.10	12.29
3.0	0.25	0.5	0.013	2.05	6.17
3.0	0.37	0.5	0.013	1.38	4.14
3.0	0.50	0.5	0.013	1.03	3.08
3.0	0.62	0.5	0.013	0.83	2.48
3.0	0.75	0.5	0.013	0.68	2.06
3.0	1.00	0.5	0.013	0.51	1.75
3.7	0.59	0.5	0.013	0.87	3.21
4.0	0.125	0.5	0.013	4.08	16.36
4.0	0.25	0.5	0.013	2.06	8.22
4.0	0.37	0.5	0.013	1.38	5.52
4.0	0.50	0.5	0.013	1.03	4.10
4.0	0.62	0.5	0.013	0.82	3.30
4.0	0.75	0.5	0.013	0.68	2.74
4.0	1.00	0.5	0.013	0.52	2.05
4.7	0.55	0.5	0.013	0.93	4.31
4.8	0.50	0.5	0.013	1.03	4.97
6.0	0.125	0.5	0.013	4.10	24.58
6.0	0.25	0.5	0.013	2.05	12.32
6.0	0.50	0.5	0.013	1.03	6.16
6.0	0.75	0.5	0.013	0.68	4.11
6.0	1.00	0.5	0.013	0.51	3.08
7.6	0.50	0.5	0.013	1.03	7.78
8.0	0.42	0.5	0.013	1.23	9.90
10.0	0.125	0.5	0.013	4.09	40.85
10.0	0.25	0.5	0.013	2.05	20.51
10.0	0.50	0.5	0.013	1.03	10.27
10.0	0.75	0.5	0.013	0.68	6.84
10.0	1.00	0.5	0.013	0.51	5.12
11.6	0.28	0.5	0.013	1.86	21.54
15.0	0.125	0.5	0.013	4.08	61.18
15.0	0.25	0.5	0.013	2.05	30.79
15.0	0.50	0.5	0.013	1.03	15.41
15.0	0.75	0.5	0.013	0.68	10.26
15.0	1.00	0.5	0.013	0.51	7.70
30.0	0.125	0.5	0.013	4.03	120.36
30.0	0.25	0.5	0.013	2.05	61.56
30.0	0.50	0.5	0.013	1.03	30.72

30.0	0.75	0.5	0.013	0.68	20.52
30.0	1.00	0.5	0.013	0.51	15.40
100.0	0.125	0.5	0.013	4.16	404.14
100.0	0.25	0.5	0.013	2.05	205.13
100.0	0.50	0.5	0.013	1.02	102.52
100.0	0.75	0.5	0.013	0.68	68.41
100.0	1.00	0.5	0.013	0.52	51.26

b. Potassium-based mixtures

**Label**		**Molar composition**			
**H_2O/KOH**	**[SiO_2+Al_2O_3]/KOH**	**SiO_2**	**Al_2O_3**	**KOH**	**H_2O**
3.0	0.125	0.5	0.013	4.10	12.29
3.0	0.25	0.5	0.013	2.05	6.17
3.0	0.50	0.5	0.013	1.03	3.08
3.0	0.62	0.5	0.013	0.83	2.48
3.0	0.75	0.5	0.013	0.68	2.06
3.0	1.00	0.5	0.013	0.51	1.75
3.3	0.59	0.5	0.013	0.88	2.89
3.6	0.55	0.5	0.013	0.94	3.41
4.0	0.125	0.5	0.013	4.08	16.36
4.0	0.25	0.5	0.013	2.06	8.22
4.0	0.37	0.5	0.013	1.38	5.52
4.0	0.50	0.5	0.013	1.03	4.10
4.0	0.62	0.5	0.013	0.82	3.30
4.0	0.75	0.5	0.013	0.68	2.74
4.0	1.00	0.5	0.013	0.52	2.05
4.5	0.47	0.5	0.013	1.10	4.99
5.2	0.43	0.5	0.013	1.21	6.25
6.0	0.125	0.5	0.013	4.10	24.58
6.0	0.25	0.5	0.013	2.05	12.32
6.0	0.38	0.5	0.013	1.36	8.17
6.0	0.50	0.5	0.013	1.02	6.15
6.0	0.75	0.5	0.013	0.68	4.11
6.0	1.00	0.5	0.013	0.51	3.08
7.0	0.31	0.5	0.013	1.64	11.42
8.3	0.23	0.5	0.013	2.20	18.21
10.0	0.125	0.5	0.013	4.09	40.85
10.0	0.25	0.5	0.013	2.05	20.51
10.0	0.50	0.5	0.013	1.03	10.27
10.0	0.75	0.5	0.013	0.68	6.84
10.0	1.00	0.5	0.013	0.51	5.12
15.0	0.125	0.5	0.013	4.08	61.18

15.0	0.25	0.5	0.013	2.05	30.79
15.0	0.50	0.5	0.013	1.03	15.41
15.0	0.75	0.5	0.013	0.68	10.26
15.0	1.00	0.5	0.013	0.51	7.70
30.0	0.125	0.5	0.013	4.03	120.36
30.0	0.25	0.5	0.013	2.05	61.56
30.0	0.50	0.5	0.013	1.03	30.72
30.0	0.75	0.5	0.013	0.68	20.52
30.0	1.00	0.5	0.013	0.51	15.40
100.0	0.125	0.5	0.013	4.16	404.14
100.0	0.25	0.5	0.013	2.05	205.13
100.0	0.50	0.5	0.013	1.02	102.52
100.0	0.75	0.5	0.013	0.68	68.41
100.0	1.00	0.5	0.013	0.52	51.26

c. Cesium-based mixtures

**Label**		**Molar composition**			
**H_2O/CsOH**	**[SiO_2+Al_2O_3]/CsOH**	**SiO_2**	**Al_2O_3**	**CsOH**	**H_2O**
3.0	1	0.5	0.013	0.51	1.75
3.0	0.75	0.5	0.013	0.68	2.06
3.0	0.5	0.5	0.013	1.03	3.08
3.0	0.25	0.5	0.013	2.05	6.17
3.0	0.125	0.5	0.013	4.10	12.29
4.0	1	0.5	0.013	0.52	2.05
4.0	0.75	0.5	0.013	0.68	2.74
4.0	0.5	0.5	0.013	1.03	4.10
4.0	0.25	0.5	0.013	2.06	8.22
4.0	0.125	0.5	0.013	4.08	16.36
6.0	1.00	0.5	0.013	0.51	3.08
6.0	0.75	0.5	0.013	0.68	4.11
6.0	0.5	0.5	0.013	1.02	6.15
6.0	0.25	0.5	0.013	2.05	12.32
6.0	0.125	0.5	0.013	4.10	24.58
10.0	1	0.5	0.013	0.51	5.12
10.0	0.75	0.5	0.013	0.68	6.84
10.0	0.5	0.5	0.013	1.03	10.27
10.0	0.25	0.5	0.013	2.05	20.51
10.0	0.125	0.5	0.013	4.09	40.85
15.0	1	0.5	0.013	0.51	7.70
15.0	0.5	0.5	0.013	1.03	15.41
15.0	0.125	0.5	0.013	4.08	61.18
30.0	1	0.5	0.013	0.51	15.40

30.0	0.5	0.5	0.013	1.03	30.72
30.0	0.125	0.5	0.013	4.03	120.36
100.0	1	0.5	0.013	0.52	51.26
100.0	0.125	0.5	0.013	4.16	404.14

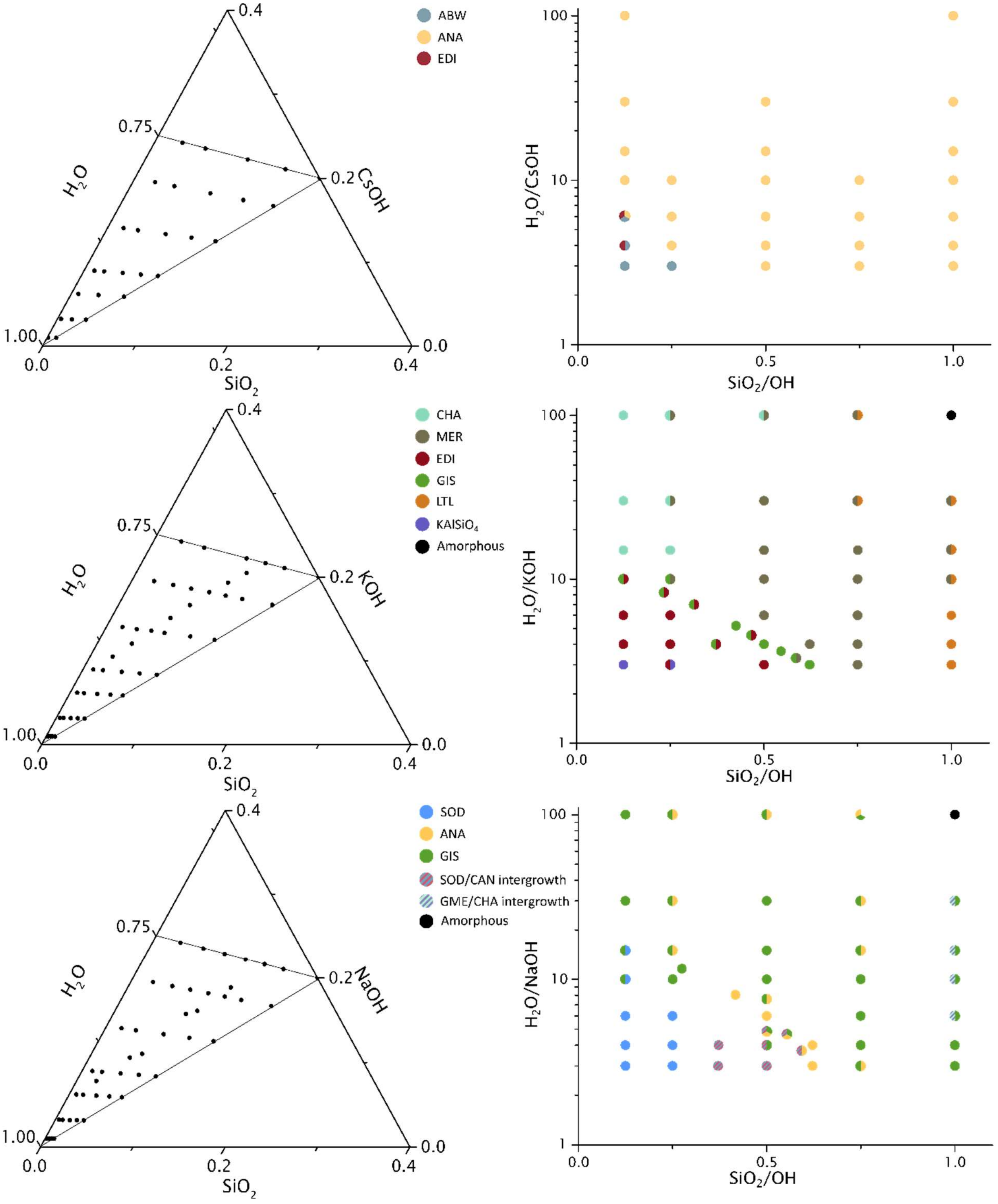

Figure S4: Synthesized batch compositions in ternary diagram representation (left) and as function of cation hydration and batch alkalinity (right).

Tabel S3: Si/Al ratio of solid samples for Na, K and Cs ternary diagrams. Empty entries indicate samples which didn't undergo chemical analysis due to lack of crystallinity or insufficient yield.

H_2O/NaOH	[SiO_2+Al_2O_3]/NaOH	Si/Al
3	1	2.25
3	0.75	1.65
3	0.5	1.11
3	0.25	1.04
3	0.125	1.00
3	0.62	
3	0.37	1.08
3.7	0.59	
4	1	2.25
4	0.75	1.71
4	0.5	
4	0.25	1.04
4	0.125	
4	0.62	1.70
4	0.37	
4.7	0.55	1.58
4.9	0.5	
6	1	2.11
6	0.75	1.66
6	0.5	1.51
6	0.25	1.02
6	0.125	0.99
7.6	0.5	
8.1	0.42	1.47
10	1	2.19
10	0.75	1.82
10	0.5	1.53
10	0.25	
10	0.125	1.43
11.6	0.28	1.26
15	1	2.18
15	0.5	1.58
15	0.125	
15	0.75	1.83
15	0.25	1.28
30	1	2.26
30	0.5	1.67
30	0.125	
30	0.75	2.01
30	0.25	
100	1	
100	0.125	
100	0.75	2.20
100	0.5	2.03
100	0.25	1.91

H_2O/KOH	[SiO_2+Al_2O_3]/KOH	Si/Al
3	1	
3	0.75	1.93
3	0.5	1.22
3	0.25	1.09
3	0.125	1.08
3	0.62	
3.3	0.59	
3.6	0.55	
4	1	
4	0.75	1.93
4	0.5	1.38
4	0.25	1.12
4	0.125	1.03
4	0.37	1.29
4	0.62	1.78
4.5	0.47	
5.2	0.43	1.44
6	1	
6	0.75	2.17
6	0.5	1.64
6	0.25	1.14
6	0.125	1.06
6	0.38	1.41
7	0.31	1.35
8.3	0.23	1.28
10	1	2.96
10	0.75	2.58
10	0.5	1.87
10	0.25	
10	0.125	1.18
15	1	2.65
15	0.5	1.95

15	0.125	1.31
15	0.75	2.25
15	0.25	1.65
30	1	2.69
30	0.5	2.07
30	0.125	1.82
30	0.75	2.59
30	0.25	
100	1	
100	0.125	
100	0.75	2.47
100	0.5	2.34
100	0.25	1.99

**H_2O/CsOH**	**[SiO_2+Al_2O_3]/CsOH**	**Si/Al**
3.00	1.00	3.97
3.00	0.75	3.44
3.00	0.50	2.30
3.00	0.25	1.33
3.00	0.125	1.14
4.00	1.00	3.89
4.00	0.75	3.2
4.00	0.50	2.44
4.00	0.25	
4.00	0.125	1.15
6.00	1.00	3.44
6.00	0.75	
6.00	0.50	2.38
6.00	0.25	1.93
6.00	0.125	1.34
10.00	1.00	
10.00	0.75	
10.00	0.50	2.63
10.00	0.25	2.01
10.00	0.125	1.76
15.00	1.00	
15.00	0.50	
15.00	0.125	1.82
30.00	1.00	3.87
30.00	0.50	
30.00	0.125	2.22
100.00	1.00	
100.00	0.125	2.32

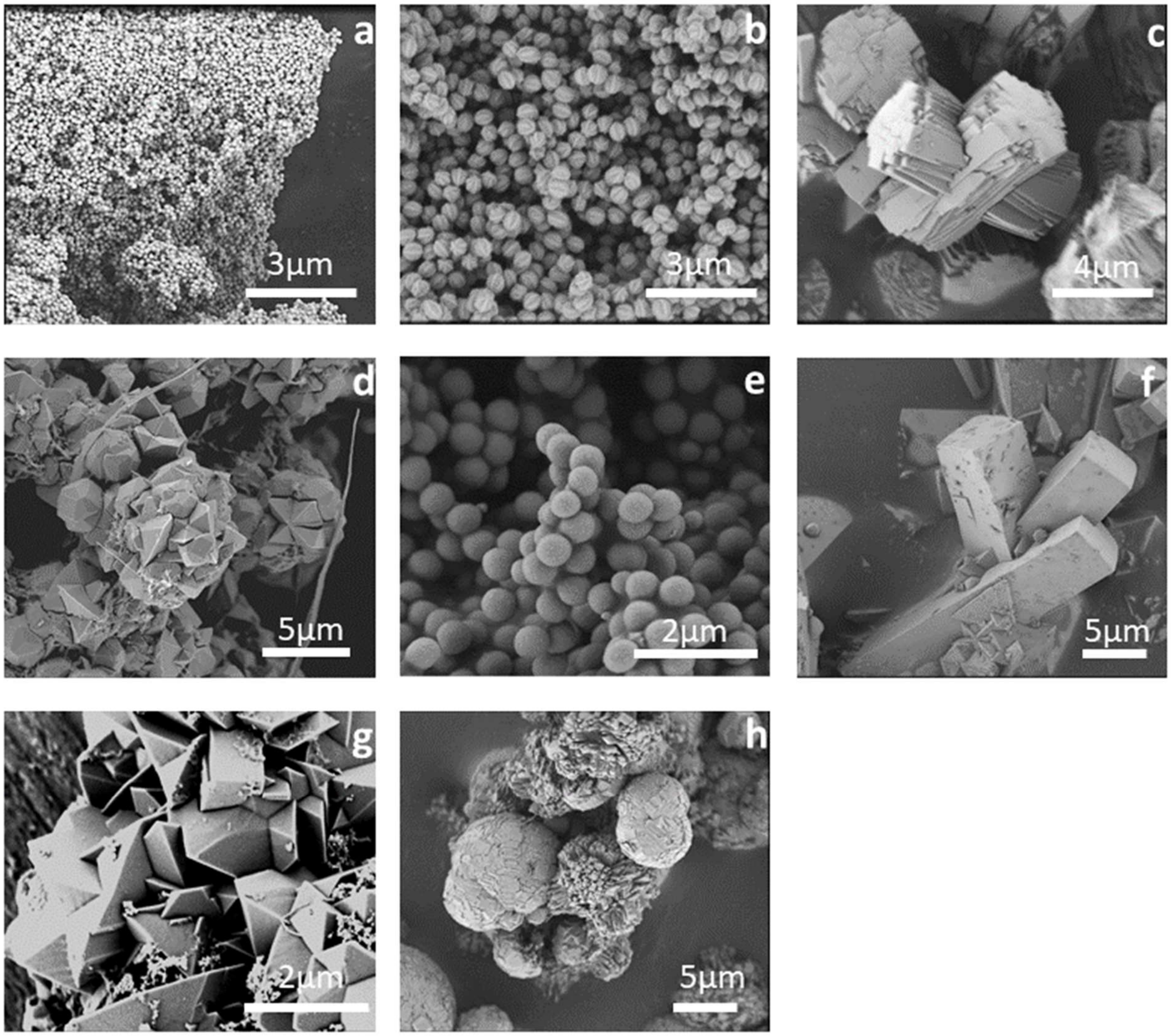

Figure S5: Selected SEM images: a) CsOH 3-1 (ANA), b) CsOH 3-0.25 (ABW), c) KOH 4-0.75 (MER), d) NaOH 3-0.375 (GIS+ANA), e) CsOH 3-0.5 (ANA), f) KOH 4-0.37 (EDI + GIS), g) KOH 4-0.5 (GIS), h) NaOH 15-1 (GME/CHA intergrowth + GIS)

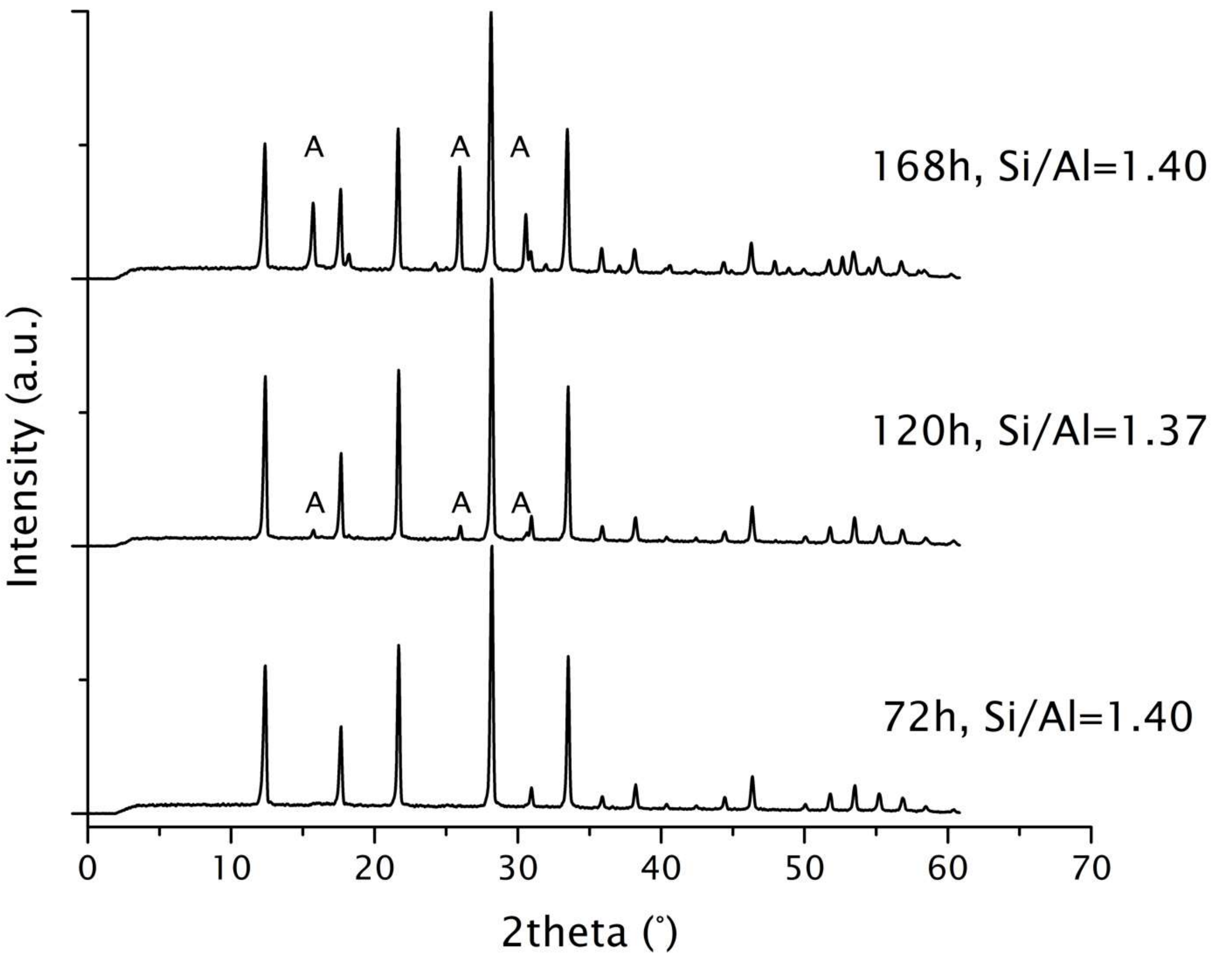

Figure S6: PXRD patterns of sample NaOH 7.6-0.5 after varying the synthesis time at 90°C. Major ANA reflections emerge over time and are indicated by the letter A.

2. Relative influence of liquid colloidal phases on zeolite synthesis

Increasing dilution or reduced alkalinity in HSIL synthesis mixtures induce formation of a colloidal phase upon addition of aluminate. To evaluate the relative importance of the liquid and solid phases on zeolite framework selection and composition, 10 representative samples that showed appreciable, visible formation of colloids were investigated. After formulation of the synthesis mixtures and aging for 24hr, the colloidal phase was removed by centrifugation yielding transparent, homogeneous supernatant mixtures which remained stable at room temperature for considerable time (days to weeks depending on the composition). ICP analysis of the removed colloidal phases (Table S4) showed they are Al-rich compared to the global synthesis mixture ($[Si/Al]_{batch}$ = 20), showing the formation of these colloids is driven by a limited solubility of aluminate at high dilution and/or limited alkalinity. Synthesis proceeded using only the liquid fractions, and the formed solids were compared to the original syntheses with the same batch composition without removal of the colloidal phase. As evidenced by the close correspondence of all diffraction patterns, synthesis with- or without the presence of colloids yielded nearly unaltered results. Two minor exceptions are the presence of a FAU impurity in NaOH 15-0.5 and a slight increase in the relative fraction of ANA to GIS in NaOH 10-0.75.

These results point out the dominant effect of the solution phase on framework selection and composition, and the presence of a low amount of aluminosilicate colloids or agglomerates is not a determinant in zeolite formation in these systems.

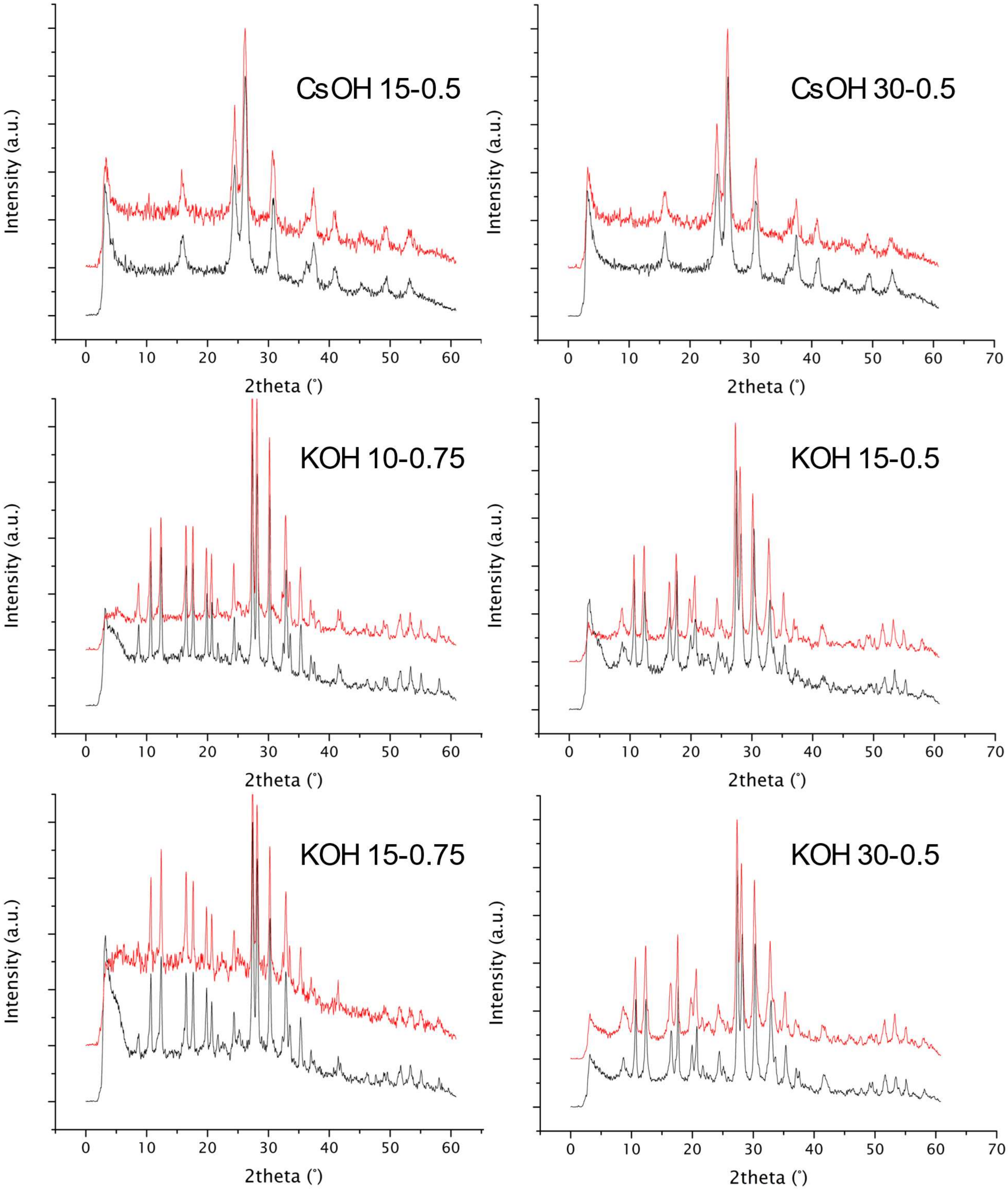
CsOH 15-0.5
CsOH 30-0.5
KOH 10-0.75
KOH 15-0.5
KOH 15-0.75
KOH 30-0.5
Intensity (a.u.)
2theta (°)
0
10
20
30
40
50
60
70

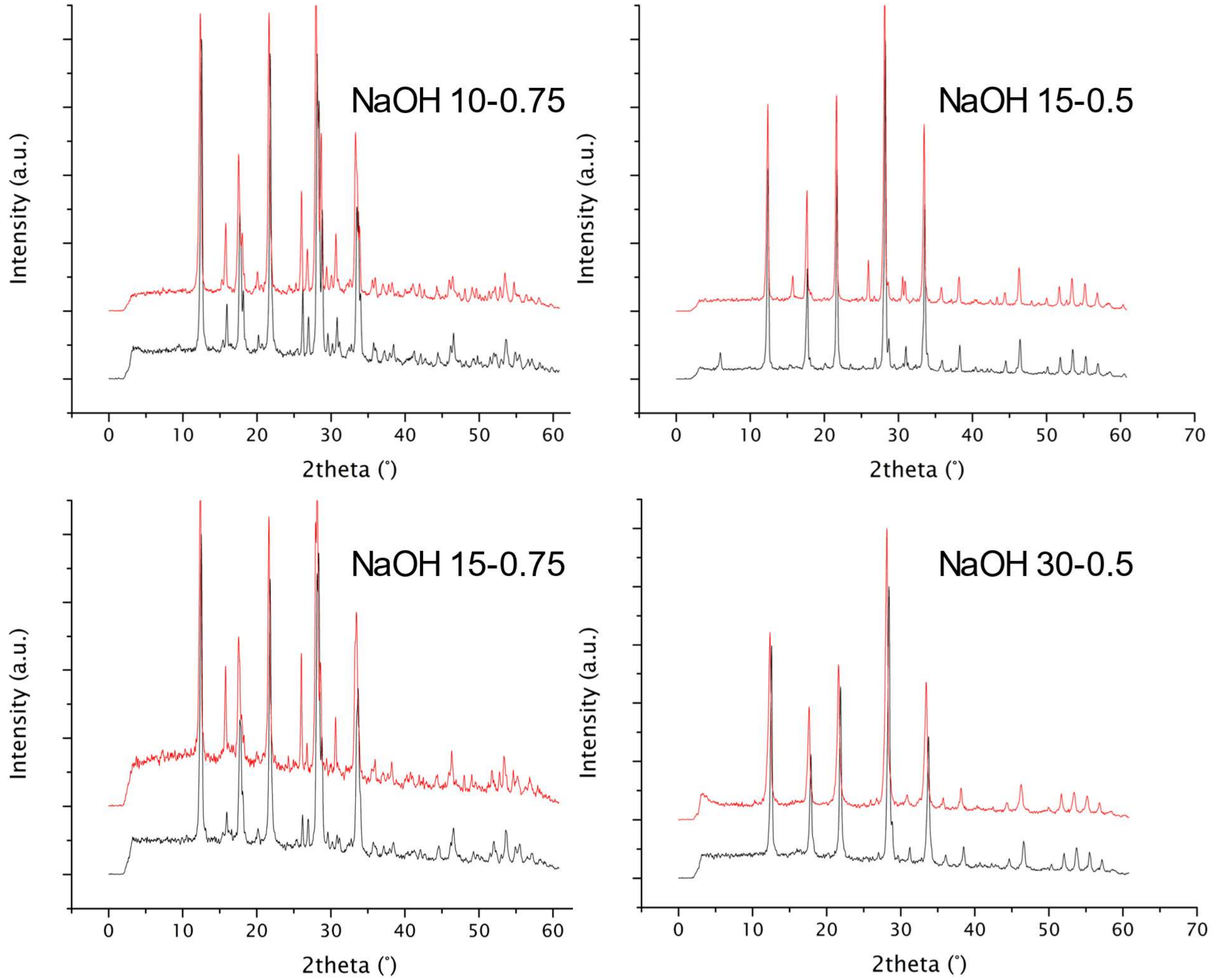

Fig. S7: XRD patterns of zeolites prepared with (black) and without (red, original syntheses) removal of colloidal fraction prior to hydrothermal synthesis.

Table S4: Si-to-Al ratio of colloidal phase separated from mother liquid after sample aging.

Sample label	Si/Al colloidal phase
NaOH 15-0.5	1.67
NaOH 30-0.5	1.84
NaOH 15-0.75	2.85
NaOH 10-0.75	3.78
KOH 15-0.5	2.11
KOH 30-0.5	1.64
KOH 15-0.75	2.32
KOH 10-0.75	2.30
CsOH 15-0.5	Insufficient material
CsOH 30-0.5	Insufficient material

Table S5: Framework Si/Al ratio of zeolites prepared with and without removal of colloidal fraction prior to hydrothermal synthesis

Sample label	original	colloid-free
NaOH 15-0.5	1.58	1.55
NaOH 30-0.5	1.67	1.72
NaOH 15-0.75	1.83	1.99
NaOH 10-0.75	1.82	1.66
KOH 15-0.5	1.95	1.81
KOH 30-0.5	2.07	2.01
KOH 15-0.75	2.25	2.20
KOH 10-0.75	2.58	2.18
CsOH 15-0.5	Insufficient yield	Insufficient yield
CsOH 30-0.5	Insufficient yield	Insufficient yield
